# The diagnostic accuracy of an initial point-of-care lactate at the emergency department as a predictor of in-hospital mortality among adult patients with sepsis and septic shock

**DOI:** 10.3389/fmed.2023.1173286

**Published:** 2023-05-24

**Authors:** Brenda Gicheru, Jasmit Shah, Benjamin Wachira, Geoffrey Omuse, Daniel Maina

**Affiliations:** ^1^Department of Pathology and Laboratory Medicine, Aga Khan University, Nairobi, Kenya; ^2^Brain and Mind Institute, Aga Khan University, Nairobi, Kenya; ^3^Department of Medicine, Aga Khan University, Nairobi, Kenya; ^4^Accident and Emergency Department, Aga Khan University, Nairobi, Kenya

**Keywords:** sepsis, septic shock, lactate, hospital mortality, outcomes, point of care systems, emergency care

## Abstract

**Background:**

In patients with sepsis, elevated lactate has been shown to be a strong predictor of in-hospital mortality. However, the optimal cutoff for rapidly stratifying patients presenting to the emergency department at risk for increased in-hospital mortality has not been well defined. This study aimed to establish the optimal point-of-care (POC) lactate cutoff that best predicted in-hospital mortality in adult patients presenting to the emergency department.

**Methods:**

This was a retrospective study. All adult patients who presented to the emergency department at the Aga Khan University Hospital, Nairobi, between 1 January 2018 and 31 August 2020 with suspected sepsis or septic shock and were admitted to the hospital were included in the study. Initial POC lactate results (GEM 3500^®^ blood gas analyzer) and demographic and outcome data were collected. A receiver operating characteristic (ROC) curve for initial POC lactate values was plotted to determine the area under the curve (AUC). An optimal initial lactate cutoff was then determined using the Youden Index. Kaplan–Meier curves were used to determine the hazard ratio (HR) for the identified lactate cutoff.

**Results:**

A total of 123 patients were included in the study. They had a median age of 61 years [interquartile range (IQR) 41.0–77.0]. Initial lactate independently predicted in-hospital mortality [adjusted odds ratio (OR) 1.41 95% confidence interval (CI 1.06, 1.87) *p* = 0.018]. Initial lactate was found to have an area under the curve (AUC) of 0.752 (95% CI, 0.643 to 0.86). Additionally, a cutoff of 3.5 mmol/L was found to best predict in-hospital mortality (sensitivity 66.7%, specificity 71.4%, PPV 70%, NPV 68.2%). Mortality was 42.1% (16/38) in patients with an initial lactate of ≥ 3.5 mmol/L and 12.7% (8/63) in patients with an initial lactate of <3.5 mmol/L (HR, 3.388; 95% CI, 1.432–8.018; *p* < 0.005).

**Discussion:**

An initial POC lactate of ≥ 3.5 mmol/L best predicted in-hospital mortality in patients presenting with suspected sepsis and septic shock to the emergency department. A review of the sepsis and septic shock protocols will help in the early identification and management of these patients to reduce their in-hospital mortality.

## 1. Introduction

The global epidemiological burden of sepsis is difficult to ascertain in part due to challenges in clinical definitions and heterogeneity in sepsis coding and reporting in clinical databases (1). There is also a paucity of data due to critical shortages in healthcare workers and a lack of access to laboratory facilities, especially in sub-Saharan Africa (SSA) (2). In Kenya, there is heterogeneity in case definitions of sepsis with prevalence ranging from 10% in adults with suspected sepsis in critical care units to 23.9% in neonatal sepsis (3, 4).

Sepsis is a clinical syndrome rather than a specific illness. This confers a diagnostic challenge given the variability in clinical signs and symptoms as well as the lack of an agreed-upon standard diagnostic test (5). One of the biomarkers featured in the sepsis guidelines is the measurement of lactate (6). Lactate levels have been long associated with tissue hypoperfusion, thus its incorporation into the clinical definitions of formerly severe sepsis and septic shock. There is now evidence that shows multifactorial causes of sepsis-associated hyperlactatemia including accelerated aerobic glycolysis, cytopathic hypoxia, direct mitochondrial impairment, and dysfunction of hepatic lactate clearance. In addition, lactate levels have been shown to strongly predict in-hospital mortality in patients with sepsis and septic shock (7–10).

There are several obstacles to the rapid determination of lactate levels in the emergency department in low- and middle-income countries (LMICs). The lack of core laboratory services may play a role in the underutilization of lactate testing in sepsis care (11, 12). In addition, prolonged turnaround time with the use of core laboratory analysis results in further limitations, as the test result must actively be sought out by the treating physician (13). Rapid testing of lactate in the emergency department in line with the sepsis bundles can be achieved with point-of-care (POC) testing, allowing for the implementation of a screening protocol in patients with suspected sepsis. POC testing allows for rapid testing with bedside results allowing for immediate intervention. It also allows for improved accuracy over core laboratory testing encumbered by pre-analytical errors such as prolonged tourniquet time or delayed centrifugation (13).

In the emergency department, lactate can be used as a marker of effective resuscitation, identification of patients with occult hypotension, risk stratification of patients, and as a mortality prediction tool (14). However, there is a lack of consensus on the optimal lactate cutoff that best predicts in-hospital mortality (15). The cutoff of 2 mmol/L is recommended by the Surviving Sepsis Campaign and incorporated into the 1 h sepsis bundle. They however caution that the recommendation is weak based on a low quality of evidence. Additionally, no studies from LMICs including SSA were included (6). There is a heterogeneity in the cutoffs proposed by different studies, using different lactate measurement platforms [central analyzers, point-of-care (POC) blood gas analyzers, handheld POC lactate devices], different patient populations, and different sample types including whole blood (arterial, venous, and capillary) and serum/plasma samples (10, 14, 16–19).

This study aimed at determining a POC blood gas lactate cutoff that best predicted in-hospital mortality in patients with suspected sepsis and septic shock presenting to the emergency department.

## 2. Methods

### 2.1. Study design and setting

We conducted a cross-sectional study at the Aga Khan University Hospital, Nairobi (AKUH, N), where medical records for patients admitted with a diagnosis of sepsis or septic shock as per the third international consensus definitions for sepsis and septic shock guidelines (SEPSIS-3) (5) criteria between 1 January 2018 and 31 August 2020 were reviewed retrospectively. Sepsis was defined as the presence of infection with signs of organ dysfunction which were represented by a Sequential Organ Failure Assessment (SOFA) score of two points or greater. Septic shock was defined as a vasopressor requirement to maintain a mean arterial pressure of 65 mm Hg or greater and a serum lactate level of >2 mmol/L in the absence of hypovolemia (5).

The Aga Khan University Hospital Nairobi Research Ethics Committee approved the study (2020-IERC/142).

### 2.2. Selection of participants

Patients with an initial POC lactate result from whole blood (arterial and venous) samples measured on the GEM 3500^®^ blood gas analyzer at admission were included. Patients with no outcome data available were excluded. All patient identifiers were removed during the data extraction process.

### 2.3. Data collection

We screened all admission records during the study period to recruit those who met the inclusion criteria. The following data were extracted using a data collection tool: patient demographic data, initial lactate result at admission from the emergency department obtained from the GEM 3500^®^ blood gas analyzer, an initial SOFA score, the focus of infection, patient comorbidities, the final diagnosis at discharge or death, and the length of hospital stay.

### 2.4. Data analysis

The data analysis was carried out using IBM Statistical Package for Social Sciences (SPSS) version 20 (IBM Corp., Armonk, N.Y., USA) software.

The study population was described using demographic, clinical, and laboratory characteristics. Descriptive quantitative variables were reported using means (± standard deviation) or medians [interquartile range (IQR)] according to their distribution. The chi-square test or Fischer's exact test compared categorical variables where appropriate.

The association between known risk factors and in-hospital mortality was determined using regression analysis. The selection of the risk factors, including age, gender, SOFA score, malignancy, and renal failure, was based on prior studies performed on patients admitted with sepsis from the emergency department. These variables were found to be associated with increased in-hospital mortality (20, 21).

A receiver operating characteristic (ROC) curve for initial POC lactate values was plotted to determine the area under the curve (AUC). The optimal lactate cutoff that best predicted in-hospital mortality was determined by calculating the Youden Index. Survival analysis was performed using Kaplan–Meier curves to determine the hazard ratio for the identified lactate cutoff. A *p*-value of < 0.05 was considered to be statistically significant.

## 3. Results

### 3.1. Demographic and clinical characteristics

A total of 159 patients were admitted to the emergency department with a diagnosis of sepsis or septic shock. Of these, 36 were excluded due to missing lactate values or incomplete outcome data. The final study cohort comprised 123 patients, and their demographic and clinical characteristics are presented in Table 1.

**Table 1:** Demographics and clinical characteristics.

	**All patients (*N* = 123)**	**Survivors (*N* = 95)**	**Non-survivors (*N* = 28)**
**Demographics:**			
Age (years) [Median (IQR)]	61.0 [41.0, 77.0]	61.0 [40.0, 75.0]	68.0 [50.0, 78.5]
**Gender**, ***n*** **(%):**			
Male	65 (52.8)	46 (48.4)	19 (67.9)
Female	58 (47.2)	49 (51.6)	9 (32.1)
**Co-morbidities [*****N*** **(%)]:**			
Diabetes	31 (25.2)	23 (24.2)	8 (28.6)
Hypertension	46 (37.4)	39 (41.4)	7 (25.0)
Renal disease	26 (21.1)	24 (25.3)	2 (7.1)
Malignancy	38 (30.9)	22 (23.2)	16 (57.1)
Neurological disorders	26 (21.1)	19 (20.0)	7 (25.0)
HIV	14 (11.4)	9 (9.5)	5 (17.9)
Pulmonary disease	4 (3.3)	3 (3.2)	1 (3.6)
Liver disease	4 (3.3)	3 (3.2)	1 (3.6)
**Focus of infection found [*****N*** **(%)]:**			
Yes	94 (76.4)	78 (82.1)	16 (57.1)
No	29 (23.6)	17 (17.9)	12 (42.9)
**Site of infection [*****N*** **(%)]:**			
Respiratory	35(37.2)	28(35.9)	7(43.8)
Renal	18 (19.1)	17 (21.8)	1 (6.2)
Bloodstream	14 (14.9)	12 (15.4)	2 (12.5)
Abdominal	14 (14.9)	12 (15.4)	2 (12.5)
Skin/soft tissue infections	12 (12.8)	8 (10.3)	4 (25.0)
Central nervous system	1 (1.1)	1 (1.3)	0 (0.0)
SOFA score [Median (IQR)]:	5.0 [3.0,7.0]	4.0 [3.0, 6.0]	7.0 [4.0, 9.5]
**Length of hospital stay:**			
Length of stay in hospital (days) [Median (IQR)]	7.0 [4.0, 13.0]	7.0 [4.0, 14.0]	8.0 [2.0, 14.0]
**Lactate:**			
Initial lactate [Median (IQR)]	3.0 [2.0, 5.0]	2.0 [2.0, 4.0]	4.0 [3.0, 8.5]

The median age of the patients was 61 years (IQR 41.0–77.0), and more than half (52.8%) of the patients were male. The most common co-morbidity was hypertension in 46 patients (37.4%) followed by malignancy in 38 patients (30.9%). The all-cause mortality rate was 22.8% (95% CI: 15.4–30.2%). Those who died had a longer median length of hospital stay, but this was not statistically significant [8.0 (2.0, 14.0) vs. 7.0 (4.0, 14.0), *p* = 0.901].

The focus of infection was identified in 94 patients (76.4%) with these patients having a better survival compared to the “no focus” group, 82.1 vs. 42.9%, respectively (*p* = 0.011). The most common site of infection was the respiratory system (37.2%) followed by the renal system (19.1%). However, there was no statistically significant difference in survival based on the sites of infection (*p* = 0.503).

Increasing SOFA scores were associated with poorer outcomes with a median SOFA score of 7.0 [4.0, 9.5] in the non-survivors compared to 4.0 [3.0, 6.0] in the survivors (*p* = 0.004).

### 3.2. Initial POC lactate

Initial lactate values ranged between 0.5 and 15 mmol/L with the median initial lactate at presentation being 3.0 mmol/L (IQR 2.0–5.0).

Median initial lactate values were higher in the non-survivors compared to the survivors, 4 mmol/L (IQR 3.0–8.5) vs. 2 mmol/L (IQR 2.0–4.0), *p* < 0.001.

After adjusting for age, gender, renal disease, malignancy, and SOFA scores, initial POC lactate was independently associated with increased in-hospital mortality [OR 1.41 95% CI (1.06, 1.87) *p* = 0.018], as shown in Table 2.

**Table 2:** Associations between risk factors and in-hospital mortality.

	**Univariate analysis**		**Multivariate analysis**	
	**Unadjusted OR (95% CI)**	***p*-value**	**Adjusted^a^ OR (95% CI)**	***p*-value**
Age	1.01 (0.99–1.03)	0.340	1.01 (0.98–1.04)	0.523
Gender (male)	2.27 (0.93–5.56)	0.074	1.79 (0.48–6.25)	0.401
SOFA score	1.22 (1.07–1.39)	0.004	1.21 (0.97–1.52)	0.093
Malignancy	4.15 (1.69–10.16)	0.002	4.19 (1.24–14.20)	0.021
Renal disease	0.22 (0.05–1.03)	0.055	0.05 (0.004–0.61)	0.019
Initial lactate	1.41 (1.16–1.71)	< 0.001	1.41 (1.06–1.87)	0.018

^**a**^Adjusted for age, gender, SOFA score, malignancy, and renal disease.

### 3.3. Optimal lactate cutoff that best predicted in-hospital mortality

**Figure 1:**
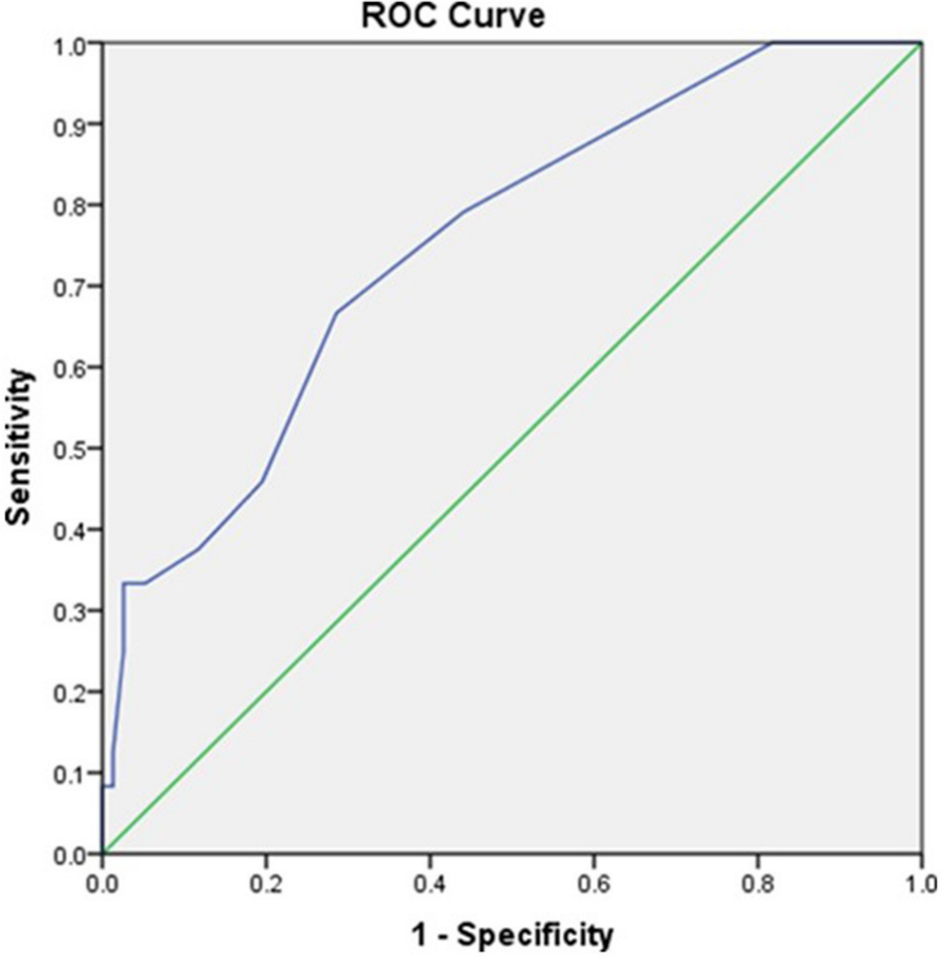
The receiver operator curve addressing the association between initial POC GEM lactate values and mortality (AUC = 0.752).

**Figure 2:**
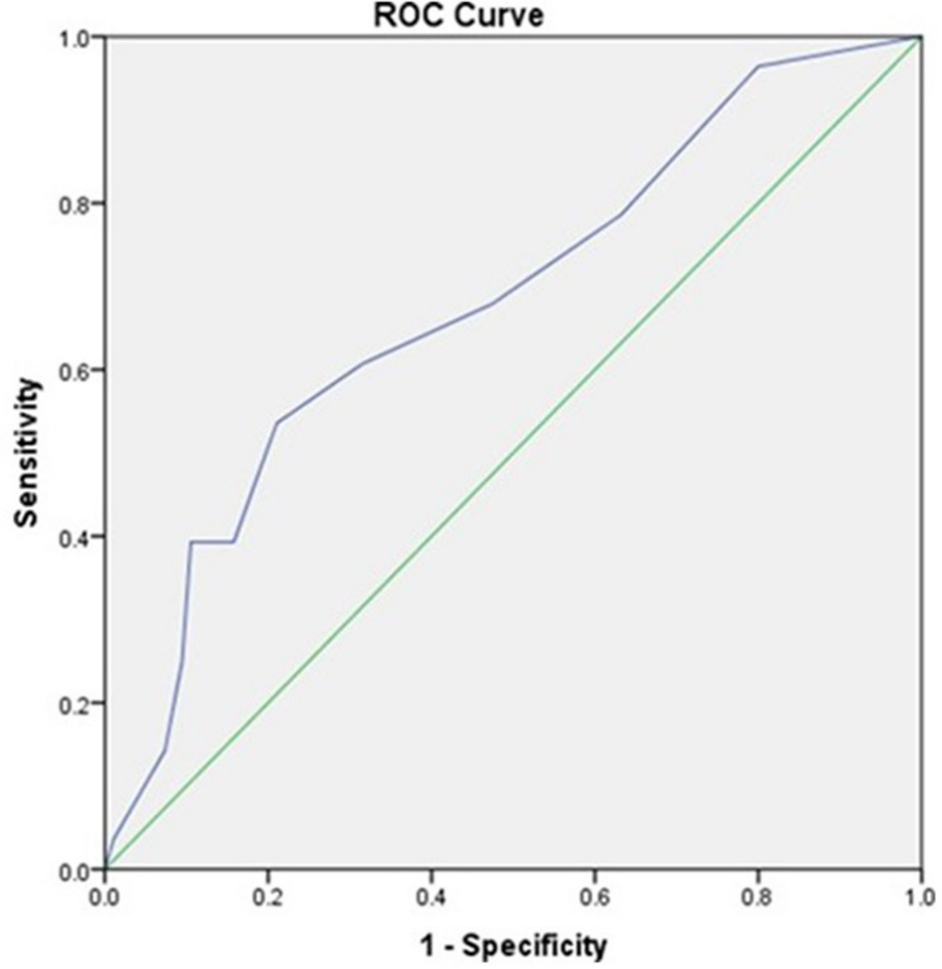
The receiver operator curve addressing the association between SOFA Scores and mortality (AUC = 0.680).

Initial lactate was found to have an area under the curve (AUC) of 0.752 (95% CI, 0.643 to 0.86) comparable to SOFA scores with AUC of 0.680 (95% CI, 0.565–0.794) as shown in Figures 1, 2, respectively.

An initial lactate cutoff of 3.5 mmol/L was found to best predict in-hospital mortality with a sensitivity and specificity of 66.7 and 71.4%, respectively, as shown in Table 3. The positive predictive value and negative predictive values were 70 and 68.2%, respectively. A cutoff of 2 mmol/L was found to have a lower specificity at 55.8%.

**Table 3:** POC lactate cutoffs and predicting for in-hospital mortality.

**Lactate score**	**Sensitivity**	**1-Specificity**	**Specificity**	**Youden Index**
0.5	1	0.987	0.013	0.013
1.5	1	0.818	0.182	0.182
2.5	0.792	0.442	0.558	0.35
3.5	0.667	0.286	0.714	0.381
4.5	0.458	0.195	0.805	0.263
5.5	0.375	0.117	0.883	0.258
6.5	0.333	0.052	0.948	0.281
7.5	0.333	0.026	0.974	0.307

**Figure 3:**
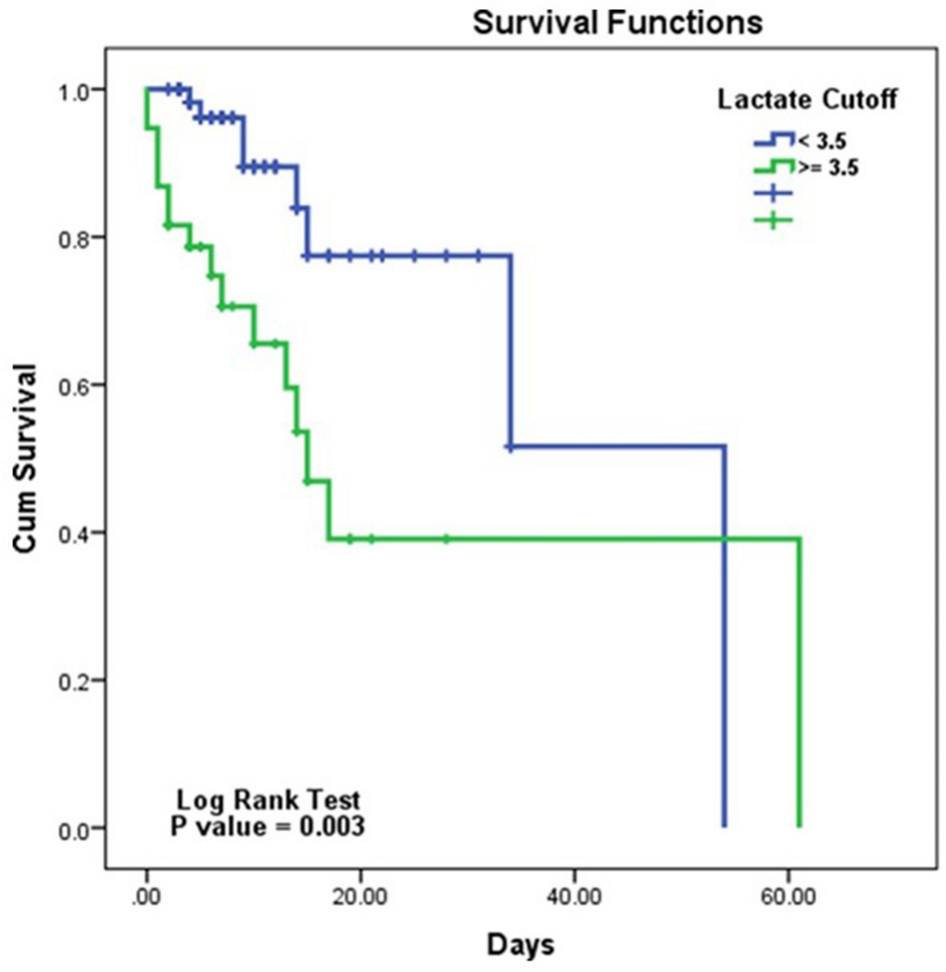
Kaplan–Meier survival curves for overall survival according to the lactate cutoff.

Mortality was 42.1% (16/38) in patients with initial lactate of ≥ 3.5 mmol/L and 12.7% (8/63) in patients with initial lactate of < 3.5 mmol/L (HR, 3.388; 95% CI, 1.432–8.018; *p* < 0.005) (Figure 3).

## 4. Discussion

In this study, non-survivors had higher lactate values than survivors. Furthermore, a lactate cutoff of 3.5 mmol/L exhibited the highest diagnostic accuracy for predicting overall in-hospital mortality based on the AUC. Patients with a lactate greater than or equal to 3.5 mmol/L had an in-hospital mortality rate 3.4 times higher than patients with a lactate < 3.5 mmol/L.

A cutoff of 2 mmol/L is part of the surviving sepsis campaign's 1 h sepsis bundle and is used to identify those patients with increased mortality risk requiring subsequent lactate measurement and close follow-up. However, the recommendation for the use of a 2 mmol/L cutoff is based on studies using different patient populations, different sample types (arterial, venous, and capillary), and different platforms (15). In our study, a cutoff of 2 mmol/L was found to have a lower specificity in predicting in-hospital mortality compared to a cutoff of 3.5 mmol/L (55.8 vs. 71.4%).

In a retrospective study by Elhouni et al. in South Africa, they evaluated the optimal lactate cutoff for predicting in-hospital mortality in patients admitted with septic shock in the critical care unit. The admission lactate was measured on a blood gas analyzer (GEM 3000). They found an initial lactate cutoff of 4.5 mmol/l to be the most optimal independent predictor of mortality (OR 2.26, 95% CI 1.14–4.52, *p* = 0.020) with an AUC of 0.612 (95% CI 0.527–0.696) (17). Although they used a similar platform to our study, they only evaluated the optimal lactate cutoff in patients with septic shock. Additionally, they had a younger cohort of patients with a median age of 42 years compared to 61 years in this study.

In a prospective cohort study in Uganda, a POC lactate cutoff was determined in HIV-positive patients presenting to the emergency department with sepsis. This was measured on capillary whole blood using a portable handheld POC lactate device (Accutrend portable lactate analyzer). A lactate cutoff of 4.0 mmol/L was identified to have the highest AUC at 0.81 in predicting in-hospital mortality in this cohort of patients (18).

In a prospective cohort study in Tanzania, a POC lactate cutoff was determined in unselected critically ill patients including patients with suspected sepsis presenting to the emergency department. This was venous whole blood tested on a portable cartridge-based POC lactate device (iSTAT Abbott). A lactate cutoff of greater than or equal to 3.8 mmol/L was found to have the highest AUC at 0.80 in predicting in-hospital mortality (19).

The differences in the lactate cutoffs may be due to the different study cohorts. Additionally, the analytical method used may impact the result given the lack of method standardization (22).

The prognostic accuracy of an initial lactate result and SOFA scores were found to be comparable in this study [AUC 0.752, 95% CI (0.643 to 0.86) vs. AUC 0.680, 95% CI, (0.565–0.794)]. This finding was similar to a retrospective study carried out by Liu et al. in China in which they found lactate AUC to be comparable to that of SOFA in patients with sepsis [AUC 0.664, (95% CI, 0.639–0.689) vs. AUC 0.686, (95% CI, 0.661–0.710)] (23). Similarly, in a retrospective study in patients with sepsis secondary to community-acquired pneumonia, the prognostic accuracy of admission lactate was comparable to that of the SOFA score [AUC 0.679, 95% CI (0.612–0.745) vs. AUC 0.795, 95% CI (0.740–0.850)] (24).

There has been a push to enhance the clinical characterization of sepsis in the absence of a gold standard diagnostic test. The SOFA score has been widely validated as the clinical operationalization of sepsis. Lactate is not part of the SOFA score but is used in sepsis algorithms to aid in risk stratification. From this study, initial POC lactate independently predicts in-hospital mortality. Additionally, a single reading of POC lactate is comparable to the SOFA score in predicting in-hospital mortality. This offers an advantage in that a lactate result can be obtained rapidly with a fast turnaround time allowing for the implementation of the 1 h sepsis bundle (6). Conversely, SOFA score parameters are time-consuming and require a well-equipped laboratory making it difficult to use in contact with patients presenting to the emergency department with sepsis (12). With this in mind, Quick SOFA (qSOFA) was created and noted to have a predictive validity similar to that of the SOFA score out of the critical care setting (5). In our facility, SOFA and not qSOFA are used in the sepsis protocol, and therefore, qSOFA was not evaluated (20).

The most common comorbidity in this cohort of patients was hypertension followed by malignancy and diabetes. Patients with underlying malignancies had overall poorer survival. This was in keeping with a prevalence study carried out by Rhee et al. in a study cohort of sepsis patients from six US hospitals where the most common underlying comorbidity was malignancy which was associated with poorer survival (25). Malignancy as a risk factor for poor outcomes in sepsis is well established due to the underlying immunosuppression (26).

Patients in whom a focus of infection was identified had a better outcome compared to those in whom a focus was not identified [82.1 vs. 42.9%, respectively (*p* = 0.011)]. However, of the 29 patients in whom an outcome was not identified, 55% had an underlying malignancy. This may have resulted in the overall poor survival of these patients. One of the cornerstones of the management of patients with sepsis and septic shock is controlling the source of infection (27). However, this can be challenging in part due to culture-negative sepsis. In a retrospective single-center study in the US, of the patients admitted with sepsis or septic shock over a 7-year period, 89% had culture-negative sepsis (28).

This study has several limitations. This was a single-center study in a private tertiary facility and thus may not be generalizable to other hospitals in Kenya, given possible differences in patient characteristics, management, and availability of resources.

The HR for the identified cutoff had a broad confidence interval. This was due to the small sample size obtained for the study. Additionally, the cutoff identified is valid only for whole blood lactate measured on a GEM blood gas analyzer. Since this measurement is not standardized, it limits the usefulness of the cutoff in sites not using the GEM instrument.

Data were not collected on whether any patient management, such as intravenous fluids or administration of antibiotics, was performed before obtaining the POC GEM lactate result, as this could alter baseline lactate values. However, our data provide evidence from a real-life setting where lactate levels are used to make timely decisions on the management of patients suspected to have sepsis.

Another limitation is that we only focused on in-hospital mortality and did not have data on the outcome of discharged patients including re-admission or transfer to other hospitals. However, mortality as an endpoint is a good indicator of the potential clinical impact of an intervention.

A further limitation is the possible reagent lot-to-lot variability with POC GEM lactate testing. However, any major variability in reagent performance would have been picked up by the daily internal quality control as well as the external quality assurance done periodically.

## 5. Conclusion

Initial lactate was found to independently predict in-hospital mortality and was found to be comparable to a SOFA score. An initial POC lactate of 3.5 mmol/L best predicted in-hospital mortality in this cohort of patients. This lactate cutoff will be a useful bedside tool for screening and rapidly stratifying patients with suspected sepsis presenting at the emergency department at risk for adverse outcomes. A review of the sepsis and septic shock protocols may help in the early identification and management of these patients to reduce in-hospital mortality at AKUHN.

## Data availability statement

The raw data supporting the conclusions of this article will be made available by the authors, without undue reservation.

## Ethics statement

The studies involving human participants were reviewed and approved by Aga Khan University Hospital Institutional Scientific and Ethics Review Committee. Written informed consent for participation was not required for this study in accordance with the national legislation and the institutional requirements.

## Author contributions

BG, DM, GO, and BW conceptualized the study. BG collected data and drafted the manuscript. JS, DM, and GO conducted data analysis and interpretation. All authors read and approved the final manuscript.
